# Performance of single-photon-counting PILATUS detector modules

**DOI:** 10.1107/S0909049509009911

**Published:** 2009-04-07

**Authors:** P. Kraft, A. Bergamaschi, Ch. Broennimann, R. Dinapoli, E. F. Eikenberry, B. Henrich, I. Johnson, A. Mozzanica, C. M. Schlepütz, P. R. Willmott, B. Schmitt

**Affiliations:** a Paul Scherrer Institut, CH-5232 Villigen PSI, Switzerland; b DECTRIS Ltd, CH-5400 Baden, Switzerland

**Keywords:** hybrid pixel detector, single photon counting, energy resolution, charge sharing, dead-time, surface X-ray diffraction

## Abstract

PILATUS is a silicon hybrid pixel detector system, operating in single-photon-counting mode, that has been developed at the Paul Scherrer Institut for the needs of macromolecular crystallography at the Swiss Light Source (SLS). A calibrated PILATUS module has been characterized with monochromatic synchrotron radiation. The influence of charge sharing on the count rate and the overall energy resolution of the detector were investigated. The dead-time of the system was determined using the attenuated direct synchrotron beam. A single module detector was also tested in surface diffraction experiments at the SLS, whereby its performance regarding fluorescence suppression and saturation tolerance were evaluated, and have shown to greatly improve the sensitivity, reliability and speed of surface diffraction data acquisition.

## Abstract 2

Characterization of PILATUS single-photon-counting X-ray detector modules regarding charge sharing, energy resolution and rate capability is presented. The performance of the detector was tested with surface diffraction experiments at the synchrotron.

## 1. Introduction

Synchrotron radiation diffraction experiments require detection systems with a large dynamic range, high count-rate capability, high detection efficiency, low or zero dark noise, and a very good point spread function to achieve the best possible signal quality. In modern diffraction and diffuse scattering experiments, the information to be gleaned from weak signals, that often lie very close to much stronger signals, is becoming increasingly important, such as in small-angle X-ray scattering and in protein crystallography (Welberry & Butler, 1994 ▶; Wall *et al.*, 1997 ▶, and references therein). The problem of ‘blooming’, associated with integrating devices such as CCDs, has until now set serious practical limitations to the effective dynamic range (Gruner *et al.*, 2001 ▶).

In addition, the need to record large data sets in times short enough to avoid radiation damage (a particular problem for organic systems) means that improved sensitivity and low background noise compared with those offered by established detector systems such as scintillators, image plates and CCD arrays are becoming a prerequisite for furthering the capabilities of diffraction facilities. PILATUS is a single-photon-counting hybrid pixel detector which has been designed to meet these requirements.

The PILATUS detector system is briefly described in the next section. Its characterization using monochromatic X-rays with respect to charge sharing, energy resolution and count rate behaviour is presented in §3. We show some of the unique detector features with surface X-ray diffraction measurements of a NdGaO_3_ sample in §4.

## 2. System description

**Figure 1:**
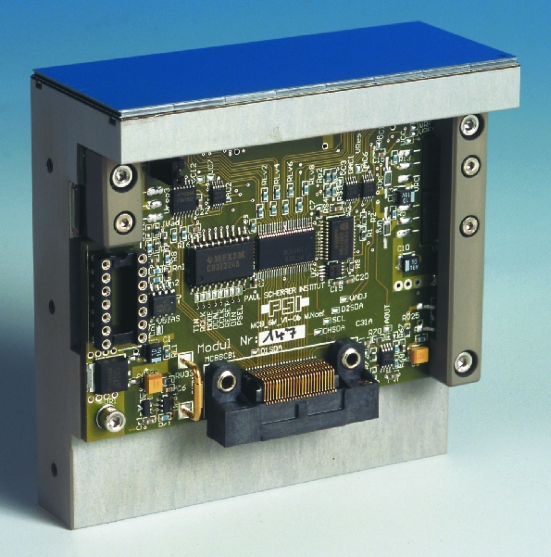
PILATUS detector module (top) on a mounting bracket (grey) with module control board (green).

PILATUS is a multi-purpose silicon hybrid pixel detector system for X-rays operated in single-photon-counting mode, purpose-built for macromolecular crystallography at the Swiss Light Source (SLS) (Broennimann *et al.*, 2006 ▶; Henrich *et al.*, 2007 ▶). A PILATUS detector module (*cf.* Fig. 1 ▶) consists of a single pixelated silicon sensor bump-bonded^1^ to an array of 8 × 2 custom-designed and radiation-tolerant CMOS readout chips (ROC). The principal specifications of the PILATUS ROC and module are listed in Table 1 ▶. The modularity of the system allows the construction of large area detectors of any array size.

**Figure 2:**
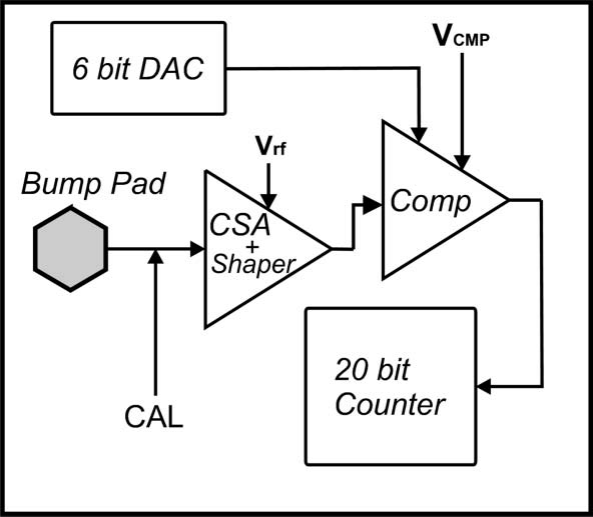
Architecture of the PILATUS ROC pixel cell.

Incident photons are directly transformed into electric charge in the silicon sensor, which is transferred *via* the bump bond to the input of the ROC pixel. A schematic of the PILATUS readout chip pixel cell is presented in Fig. 2 ▶. The analog front-end of a readout pixel consists of a charge-sensitive preamplifier (CSA) and an AC coupled shaper. The gain and shaping time of the CSA are adjusted with a global voltage (*V*_rf_). An analog pulse from the shaper is discriminated against a threshold in the comparator (Comp) after amplification. The comparator threshold of each pixel is set with a global threshold voltage (*V*_cmp_) and is further individually trimmed using an additional in-pixel 6-bit digital-to-analog converter (DAC). If the pulse amplitude exceeds the threshold, a digital signal is produced which increments the 20-bit counter. This detection principle is free of dark current and readout noise effects but requires precise calibration of the pixel threshold for optimum performance.

The amplitude and width of the analog pulses after the shaper are correlated such that larger pulses are simultaneously wider. The CSA gain settings can be selected to meet the experimental requirements regarding energy and rate of the radiation. Three different settings of *V*_rf_ were defined for the detector characterization presented here: low-gain, medium-gain and high-gain CSA settings. Each setting requires an individual global threshold calibration, threshold trimming and rate correction. The global threshold of the module was calibrated with respect to the X-ray energy, and trim files were created and applied to minimize the pixel-to-pixel threshold dispersion. The PILATUS detector system and the calibration methods have been described in detail by Kraft *et al.* (2009 ▶).

## 3. Characterization

All the characterizations presented here were carried out at the surface diffraction station of the material science beamline X04SA of the SLS (Patterson *et al.*, 2005 ▶), since better results are obtainable using monochromatic X-rays than using the internal calibration signal (CAL, *cf.* Fig. 2 ▶) of the readout chip. Either the direct synchrotron beam in combination with absorbing filters or an elastic scatterer for homogeneous detector illumination was used.

### 3.1. Threshold scan

Many detector characteristics can be derived from threshold scans. For that purpose the module is homogeneously illuminated with monochromatic X-rays. Images of equal exposure time are taken while the global threshold (*E*_th_) is increased with respect to energy for each frame.

The s-curve method (Dinapoli, 2004 ▶) is used to analyze the threshold scans. In our case the s-curve is extended by a linear factor to take into account the charge-sharing^2^ contribution. This representation of the s-curve is empirically based on observations and describes the data well. A previous energy calibration of the *V*_cmp_ bias controlling the global threshold of the module in order to convert the abscissa from voltage to energy is required,
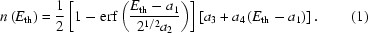
The s-curve (1) has a well defined inflection point *a*_1_ which is the threshold of the pixel for the incident X-ray energy (*E*_in_) that was used. The parameter *a*_2_ is related to the electronic noise, charge sharing and energy spectrum^3^ of the incident X-rays. The magnitude of *a*_3_ is determined by the flux of the source and the exposure time. The slope *a*_4_ in the linear term models the charge sharing of the sensor.

**Figure 3:**
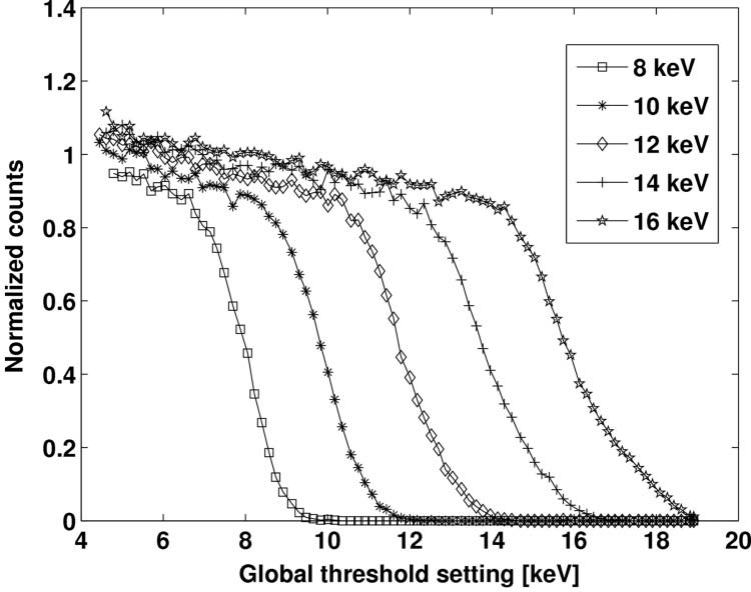
Threshold scans of a pixel with low-gain CSA settings for different incident X-ray energies.

Threshold scans of an individual PILATUS pixel for five different beam energies as a function of the global threshold are shown in Fig. 3 ▶. For the purpose of comparison, the data of each scan in Fig. 3 ▶ are normalized to the number of counts recorded with the global threshold set at 50% of *E*_in_.

### 3.2. Charge sharing

Incident photons are converted into charge clouds inside the sensor, which are transported by the applied electrical field to the collection electrodes. Owing to diffusion and Coulomb repulsion, the diameter of the charge cloud increases while it drifts towards the collection electrode. In the case of conversion close to or at the border between pixels, the signal will therefore be shared among adjacent pixels (Ponchut *et al.*, 2002 ▶; Bergamaschi *et al.*, 2008 ▶).

Charge sharing in the predecessor of the current PILATUS detector system was characterized using an infrared laser to determine the effective charge collection area (Broennimann *et al.*, 2002 ▶). Alternatively, the influence of charge sharing on the count rate can be directly derived from threshold scans (Tlustos *et al.*, 2004 ▶). However, the method of measuring the charge sharing by means of threshold scans with monochromatic X-rays is advantageous over the laser method with respect to real operation conditions, because the infrared photons convert into charge at the surface of the sensor, whereas X-rays convert throughout the bulk of the silicon.

The parameter *a*_4_ in (1) represents the slope of the linear decrease in count rate of the threshold scans occurring for global threshold settings below the incident photon energy. This slope is due to charge sharing among adjacent pixels. Since (1) describes a threshold scan in absolute counts, *a*_4_ is normalized becoming independent of exposure time and photon flux, thus thereby being comparable. For the normalization, the count *N*_50_ is used, which is registered with the global threshold set to 50% of *E*_in_. At this particular threshold setting, the count rate is independent of charge sharing and represents the number of correctly counted photons. Thus the normalized charge sharing slope is *k* = *a*_4_/*N*_50_.

Since charge sharing occurs at the borders of the sensor pixel, its effect depends on the ratio between the perimeter and pixel area. In a simple model, the normalized charge-sharing slope is associated with a corresponding fraction of area of the sensor pixel in which the charge of converting photons is shared, *e.g.* for *k* = 4.5% keV^−1^ at *E*_in_ = 12 keV the corresponding area is *k* *E*_in_/2 = 27.2% of the pixel. Regarding this area to be a strip along the pixel border, we can calculate its width. Using the example above, the strip width is 12.6 µm for a normal PILATUS pixel. Assuming the width to be the same for any pixel size, *k* can be calculated for different pixel sizes from the known *k* of a particular pixel size.

**Figure 4:**
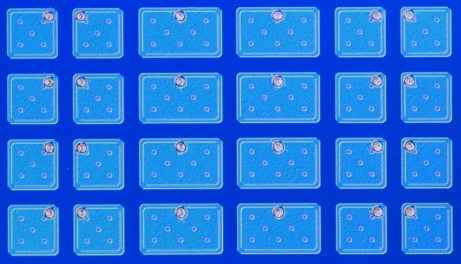
Light microscope image of the sensor flip side with normal pixels (square) and large pixels (rectangular) spanning the gaps between readout chips.

**Figure 5:**
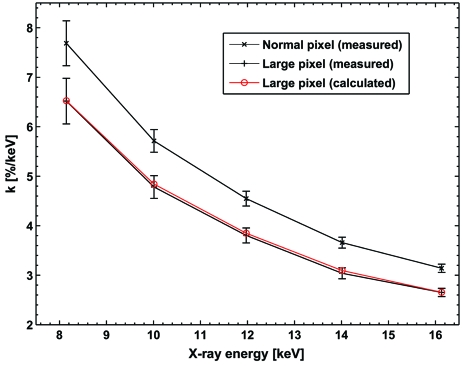
Average normalized charge-sharing slope (*k*) at different X-ray energies for normal-sized and large pixels including the calculated *k* for large pixels.

The normalized charge-sharing slopes of the PILATUS were determined from threshold scans taken with 8, 10, 12, 14 and 16 keV X-rays. The above-mentioned geometrical considerations were applied to calculate the *k* value for the large sensor pixels spanning the gaps between the readout chips^4^ (*cf.* Fig. 4 ▶) and compared with the measurements. The averaged *k* value for normal-sized pixels and the averaged *k* value for the larger pixels with the calculated *k* value according to the above model are shown in Fig. 5 ▶.

### 3.3. Energy resolution

A threshold scan curve of a pixel corresponds to the integrated energy spectrum of the X-ray source above the threshold. The electronic noise of the sensor, and the analog and digital front-end, further broaden the spectrum. Hence, the derivative of a threshold scan performed with monochromatic X-rays yields a spectral peak in which the apparent width is a measure of the energy resolution of the pixel. In addition to the resolution of a single pixel, the pixel-to-pixel threshold dispersion has to be included for the overall energy resolution (OER) of the detector system. Since noise in the threshold scan data is enhanced by numeric differentiation, several thousand incident photons per pixel are required to obtain a reliable peak. To achieve better statistics, the threshold scans of a module were averaged before differentiation, *i.e.* the average count of all pixels was calculated for each threshold value. Thereby, the threshold dispersion is also taken into account.

**Figure 6:**
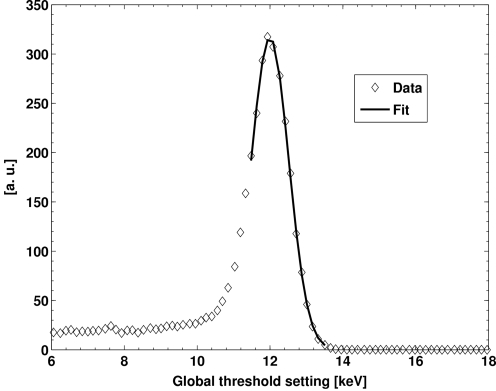
Derivative of an averaged threshold scan for 12 keV X-rays fitted with a Gaussian.

The derivative of an averaged threshold scan of a trimmed^5^ module for 12 keV X-rays is shown in Fig. 6 ▶. The constant tail towards low threshold energies stems from charge-sharing effects which only marginally affect the peak shoulder towards high energies. A Gaussian is fitted to the data in the region of the right shoulder including the peak. The OER of the system is given by the full width at half-maximum [FWHM = 2(2ln2)^1/2^σ] of the peak, where σ^2^ is the variance of the fitted Gaussian.

**Figure 7:**
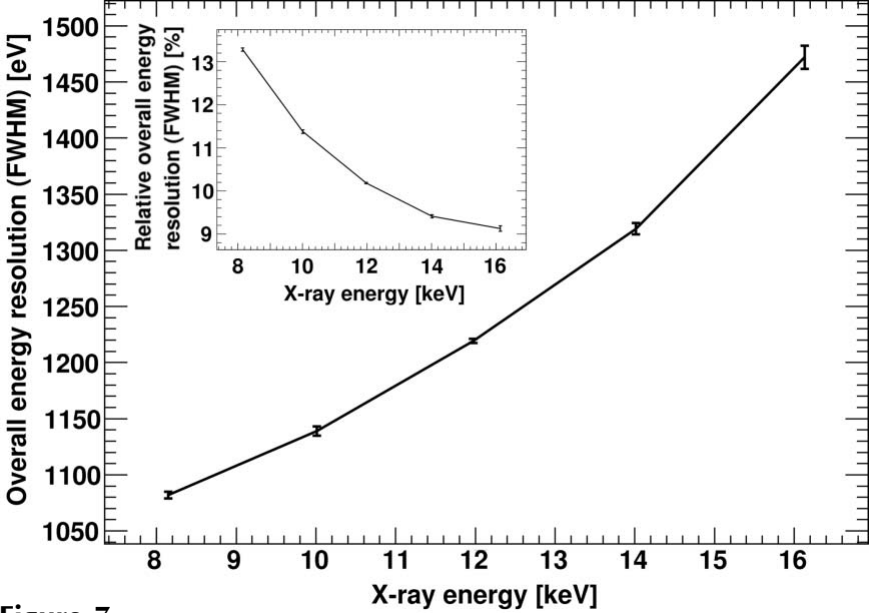
Overall energy resolution of a trimmed PILATUS module for different X-ray energies using low-gain CSA settings. Inset: relative overall energy resolution.

The PILATUS module was trimmed for three different CSA gain settings and for each setting a threshold scan was taken using 8 keV X-rays. The OERs obtained by the described method from the scans are listed in Table 2 ▶. The module was also trimmed at 8, 10, 12, 14 and 16 keV for low-gain CSA settings, and a threshold scan was recorded for each energy to study the energy dependence of the OER. The resulting OER as a function of incident X-ray energy is shown in Fig. 7 ▶.

Since electronic noise scales with the reciprocal of the CSA gain, and the achieved threshold dispersion after trimming is smaller for high-gain CSA settings (Kraft *et al.*, 2009 ▶), the OER improves with an increase in gain.

### 3.4. Dead-time

The counting behaviour of the PILATUS detector for high-intensity synchrotron beams was investigated at the surface diffraction station of the material science beamline X04SA of the SLS. The beamline features 15 filter sheets of Al, Ti and Mo of different thicknesses to control the attenuation of the beam. To avoid beam hardening owing to absorbers, the monochromator was set to 16 keV, because the higher harmonics (≥32 keV) are greatly suppressed by the lower wiggler flux at high energy, the low mirror reflectivity and the low Si scattering factors (Patterson *et al.*, 2005 ▶). The remaining radiation from higher harmonics is further suppressed, since the efficiency of the Si sensor is less than 10% above 30 keV. Therefore the use of filter sheets to create different beam intensities is justified. The transmissions of the sheets were previously calibrated using the PILATUS detector at low intensity (<100000 counts pixel^−1^ s^−1^). In doing so, the direct synchrotron beam was defocused on the module such that the spot size was several millimetres in diameter. The same set-up was used to investigate the counting behaviour of the detector. For each filter transmission which was increased in small steps, a frame was recorded. The exposure time was set to 20 ms, preventing the counter overflow at very high intensities. Data were recorded with medium- and low-gain CSA settings at *E*_th_ = 6, 8 and 10.7 keV.

The time structure of the SLS during the experiment was a flat-filled electron beam of *t*_on_ = 780 ns with 390 electron bunches of approximately 50 ps length every 2 ns, followed by a gap of 180 ns containing a single bunch. Regarding the X-ray delivery as being Poisson distributed during *t*_on_ from the detector point of view and considering the given time structure of the beam, a Monte Carlo (MC) model can be employed to simulate the response of the detector to the incident rate (Bateman, 2000 ▶).

**Figure 8:**
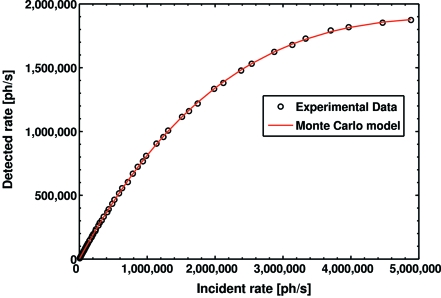
Detected count rate of a pixel as a function of the incident rate for 16 keV X-rays using 50% relative global threshold and medium-gain CSA settings. The Monte Carlo model prediction agrees well with the data.

**Figure 9:**
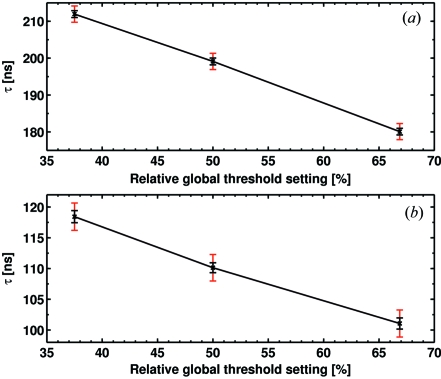
Average dead-times (τ) for 12 and 16 keV X-rays with respect to the relative global threshold settings for medium gain (*a*) and low gain (*b*) CSA settings. The black error bars represent the statistical errors on the average τ, the red error bars represent the total statistical and systematic errors.

MC data were generated for 100 different dead-times between 50 and 250 ns. The MC data for each dead-time were parameterized and the parameterizations compared with the experimental data of a pixel (*cf.* Fig. 8 ▶). By means of minimum χ^2^ between parameterization and experimental data, the corresponding dead-time (τ) was determined. The average τ, obtained in this way, with respect to the relative global threshold setting (*E*_th_/*E*_in_), are presented in Fig. 9 ▶. The shorter τ for low-gain CSA settings originates from leaner pulses entering the comparator, hence the time to resolve two successive pulses is less than for medium-gain CSA settings. Common for both CSA settings is a monotone drop in τ for increasing *E*_th_/*E*_in_. Experimental data taken at *E*_th_/*E*_in_ = 83% show a significant deviation from the Monte Carlo model at incident rates above 1 × 10^6^ photons s^−1^, meaning that pile-up effects become dominant and thus the experimental data are insufficiently described by the utilized model (Laundy & Collins, 2003 ▶).

In case of a flat-filled synchrotron beam without gap or an X-ray tube, the photon delivery in time can be considered as uniform, and the loss in counting efficiency at high rates can be calculated using the analytic model (Bateman, 2000 ▶),
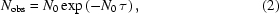
where *N*_obs_ depicts the detected rate and *N*_0_ the true incident photon rate. Therefore an offline rate correction in software can be applied. In order to minimize computing time for large series of exposures with constant exposure time (*t*), the rate correction is accomplished by means of a look-up table. A look-up table mapping recorded counts (*N*_obs_ *t*) to the corresponding incident counts (*N*_0_ *t*) using (2) is created for a specific dead-time and a specific exposure time, when either of them is changed. After an image is transferred to the data acquisition computer, the counts of each pixel are replaced according to the look-up table. The incident rate per pixel should stay at least below the detectable maximum rate given by *N*_0_max__ = 1/τ, since a higher rate becomes ambiguous in terms of the detected rate and would lead to misinterpretation. The exposure time has to be adapted to the detected rate in order to avoid overflow of the counter because the counter starts to count again from zero after its range is exceeded. Also noteworthy is the loss of statistics owing to the reduced counting efficiency at high rates, meaning that the relative statistical counting error of a pixel is given by the number of detected photons, which is larger than the same error of the number of incident photons. This leads to a decrease of the signal-to-noise ratio in the data for an increase in rate. This simple rate correction will fail if the incident rate changes during an exposure, since the correction method assumes a constant rate per pixel. In the case of the above-mentioned time structure of the SLS, the error between (2) and the Monte Carlo model is approximately 2% for an input rate of 420000 photons s^−1^ pixel^−1^ (630000 photons s^−1^ pixel^−1^) at 50% relative global threshold for low (medium) gain CSA settings.

## 4. Surface diffraction with the PILATUS 100K

A PILATUS 100K system consisting of one module has been used for surface diffraction at the material science beamline X04SA of the SLS since 2006. It was demonstrated by surface diffraction experiments that PILATUS provides data of superior quality and that measuring times are shortened owing to the fast readout (Willmott *et al.*, 2007 ▶). The diffraction signal on the 20L crystal truncation rod (CTR) of a NdGaO_3_(110) surface was measured in order to demonstrate the unique detector features of fluorescence suppression and saturation tolerance.

### 4.1. Fluorescence suppression

Setting the pixel threshold to exactly 50% of the incident photon energy results in maximum detection efficiency, while avoiding double counting owing to charge sharing (Kraft *et al.*, 2009 ▶). In some circumstances, however, it can be favourable to choose a higher threshold in order to suppress disturbing the fluorescent background at the expense of a slight decrease in efficiency (Ponchut & Zontone, 2003 ▶). In the case of NdGaO_3_, the Ga absorbs at 10.367 keV and fluoresces at 9.252 keV (*K*α_1_), 9.225 keV (*K*α_2_) and 10.264 keV (*K*β_1_), producing a significant background (Thompson *et al.*, 2001 ▶).

**Figure 10:**
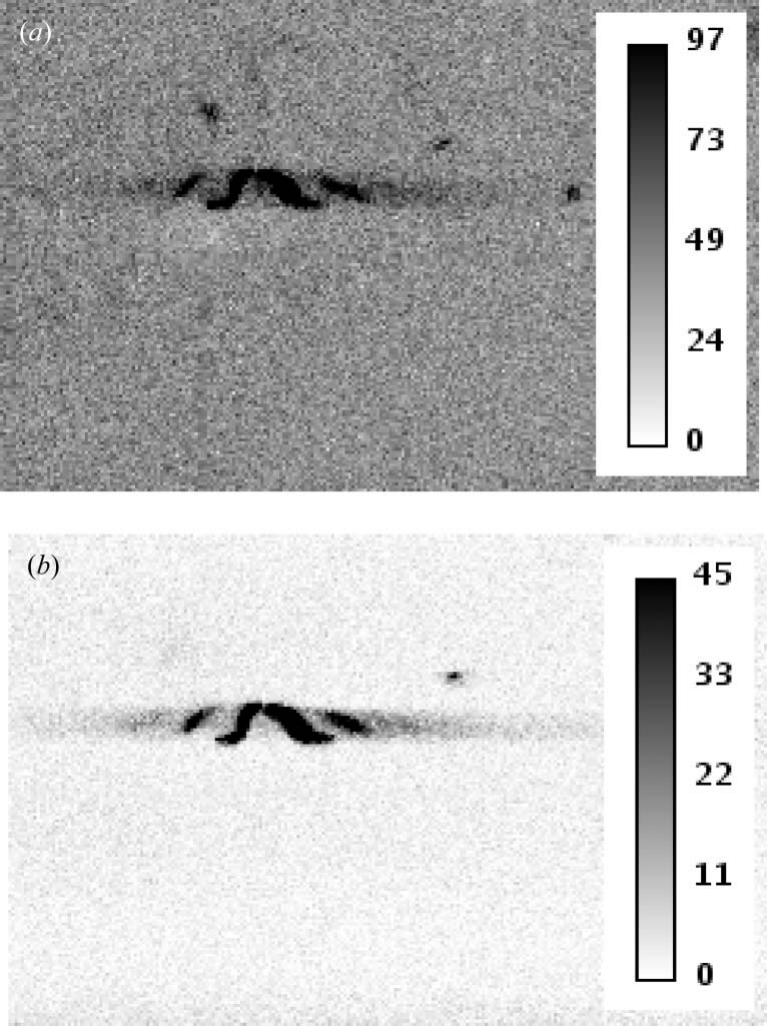
Detector image (zoom) of the 201 signal of NdGaO_3_ using 15.92 keV X-rays and a global threshold of (*a*) 9.5 keV and (*b*) 12 keV.

**Figure 11:**
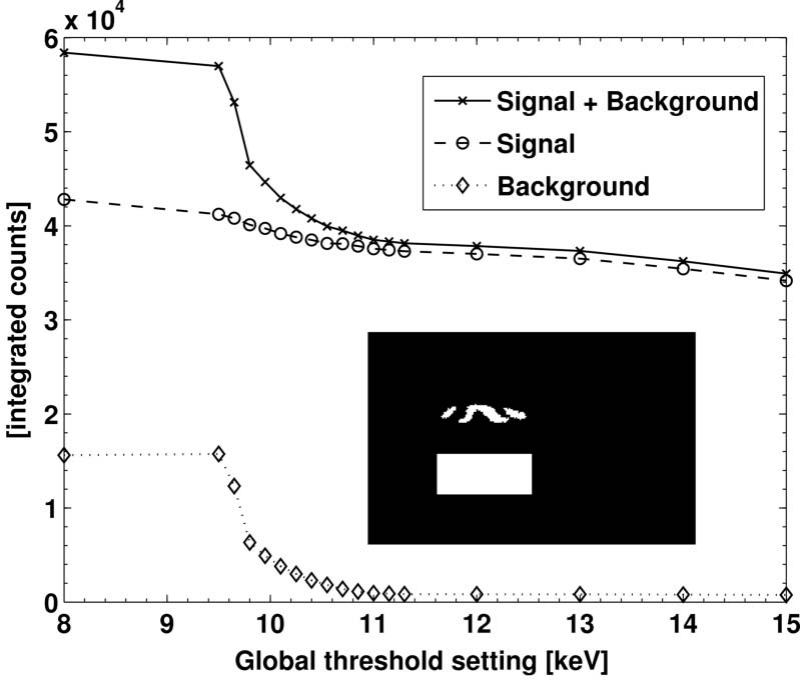
Integrated signal, integrated background and integrated signal plus background of the 201 signal of NdGaO_3_ with respect to the global threshold using 15.92 keV X-rays. Inset: the white rectangle marks the ROI where the background was determined; the white shape on top of the rectangle marks the ROI for signal integration.

To demonstrate the effect of changing the threshold levels, the incident X-ray energy was set to 15.92 keV and the detector was positioned at the (201) point in reciprocal space, which is the weakest point on the CTR in between the (200) and the (202) Bragg peaks. A series of images was recorded while increasing the global threshold between 8 and 15 keV (*cf.* Fig. 10 ▶). The total intensity of the (201) diffraction signal was obtained by summing all counts inside a region of interest (ROI) comprising those pixels containing the diffraction feature. To determine the average background, the counts of all pixels inside a second ROI in the form of a box below the signal were averaged (*cf.* inset of Fig. 11 ▶). The background below the signal is equal to the average background multiplied by the number of pixels in the signal ROI. Subtracting this background from the integrated counts in the signal ROI yields the true diffraction signal.

**Figure 12:**
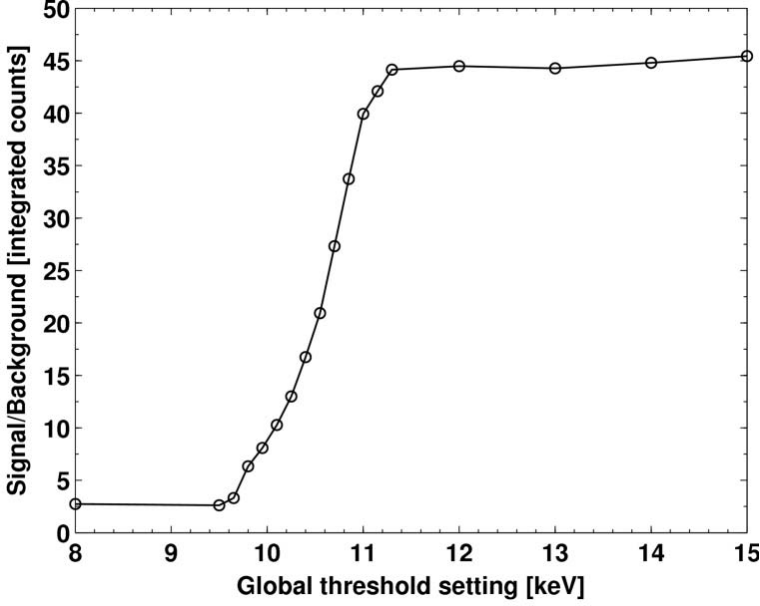
Signal-to-background ratio of the 201 signal of NdGaO_3_ with respect to the global threshold using 15.92 keV X-rays.

The integrated signal, integrated background and the total measured intensity inside the signal ROI as a function of the global threshold energy are shown in Fig. 11 ▶. The background is significantly reduced when the global threshold exceeds the fluorescence energies of the Ga, as expected. In comparison, the true diffraction signal decreases only slightly over the scanned threshold range, owing to the loss of detection efficiency. As a result, the signal-to-background ratio is strongly enhanced from 2.7 at 8 keV threshold to 44.5 at 12 keV threshold (Fig. 12 ▶).

### 4.2. Saturation tolerance

A frequent problem in diffraction experiments is the measurement of weak signals very close to strong signals. Owing to its radiation tolerant design and complete lack of blooming, the PILATUS detector allows exposures with respect to the weak signals to be optimized even if this involves complete saturation of any neighbouring strong signal. To investigate whether this is really true, or whether in fact heavy over-irradiation on one part of the detector affects other areas which are not saturated, the intensity distribution around the very intense (202) Bragg peak was recorded in two reciprocal-space scans taken with nominal filter transmission values of 1 ± 0.01 (high) and (1 ± 0.01) × 10^−2^ (low), under otherwise identical conditions.

**Figure 13:**
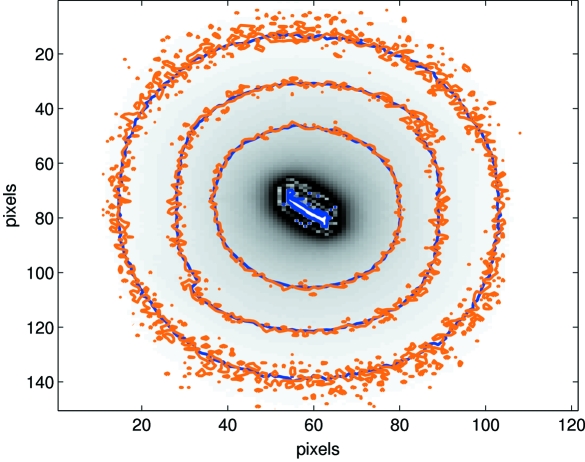
Detector image taken at the nominal Bragg peak position (shaded frame in Fig. 14 ▶). The central part is saturated to several orders of magnitude above the maximum detectable count rate. Plotted on top there are several contour lines for high (dark, blue) and low (light, orange) transmission data, respectively.

The image taken at the nominal Bragg peak position is shown in Fig. 13 ▶. The strong Bragg peak in the centre of the visible feature is heavily saturated when using the high-transmission value. Several contour lines are plotted on top of the image. Contours of 8000, 16000 and 32000 counts for the data taken with high (dark, blue) transmission are overlayed on the corresponding contour lines of the low (light, orange) transmission data (*i.e.* with 80, 160 and 320 counts).

**Figure 14:**
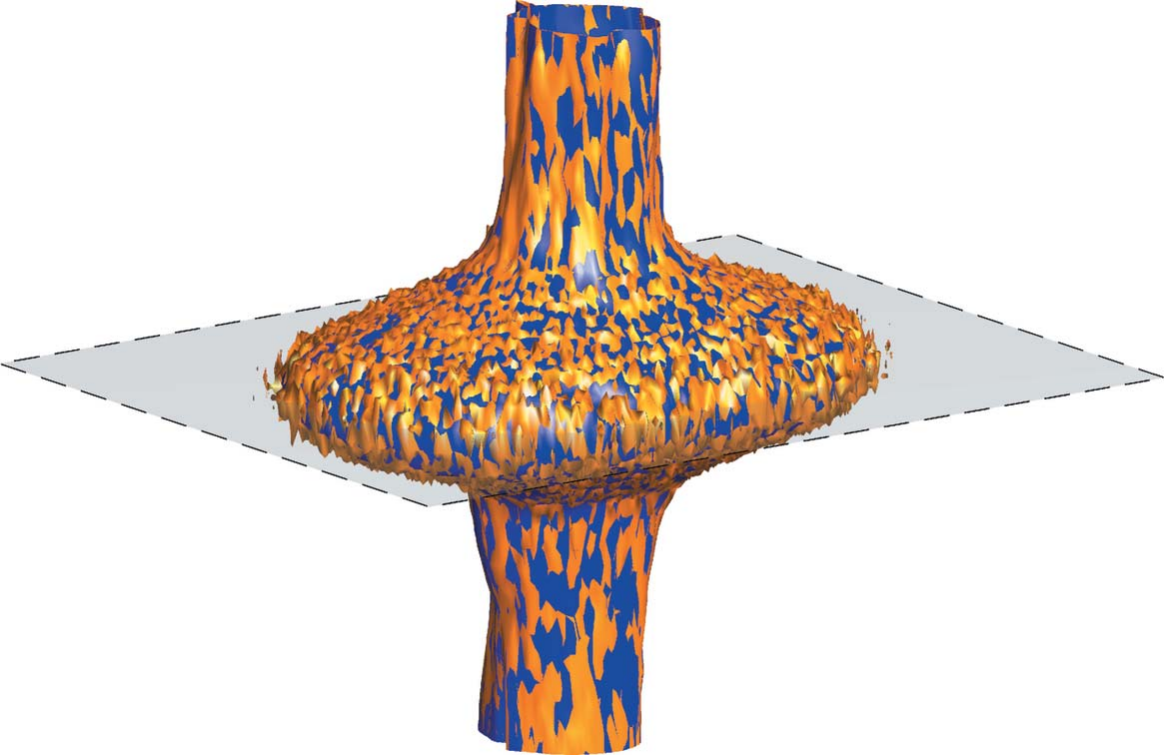
Intensity isosurfaces for 10000 (dark, blue) and 100 (light, orange) counts from data taken with filter transmissions of 1 (high) and 0.01 (low), respectively. The two isosurfaces coincide perfectly.

By stacking of the individual detector frames of each scan, artificial intensity volume data sets can be constructed.^6^ These volume data can be used to produce intensity maps or line cuts through the sampled reciprocal-space volume, or to visualize isosurfaces of the intensity distributions, as shown in Fig. 14 ▶. Here, we compare the isosurfaces for the same nominal intensities of 10000 and 10000/100 = 100 counts for high and low transmission data, respectively. The fact that the two isosurfaces coincide excellently, within the noise of the data, proves that over-irradiation in one part of the images does not affect the performance of those pixels which are not saturated.

An important consequence of this is that weak signals near strong peaks can be measured with an unattenuated beam without the strong signal either affecting the weak signal or damaging the detector. Hence there is no need to use a beam stop to obscure the strong diffraction signal (*e.g.* a Bragg peak), and simpler measuring geometries can be used which offer more flexibility, reliability and speed. One should bear in mind, however, that very strong signals such as that produced by the direct focused synchrotron beam will still cause radiation damage to the detector in a few seconds (Sobott *et al.*, 2009 ▶). In addition, it should be mentioned that the above measurements were made using a focused beam produced by a wiggler. If focused undulator radiation is used (where the focus spot is usually much smaller compared with a wiggler beam), it is possible that unattenuated Bragg peaks could damage the detector.

## 5. Conclusions and outlook

PILATUS detector modules were carefully calibrated and characterized with monochromatic X-rays. The effect of charge sharing on the count rate is relatively small and significantly decreases for higher X-ray energies. The measured normalized charge-sharing slope for the larger sensor pixels is well described by the simple geometric charge-sharing model. The presented overall energy resolution is of interest if the threshold needs to be set above a fluorescent background or if position-sensitive spectroscopy is performed by subtracting images of different thresholds.

Offline rate correction is crucial for photon-counting detectors if high rate signals are measured. Correction in case of constant rate during exposures is accomplished with an analytic model and is routinely applied by the PILATUS detector systems. If rate fluctuations owing to a gapped synchrotron beam or changes in an investigated sample occur, this issue becomes more complex and requires special treatment depending on the reason for the fluctuations. Owing to the modularity of the PILATUS system, all of the reported methods and results also apply for large arrays of modules. Detectors with arrays of 60 modules (PILATUS 6M) and of 24 modules (PILATUS 2M) were realised. These detectors were calibrated by the same methods as the single module detector (PILATUS 100K) and are successfully operated at beamlines of the SLS.

It was verified in surface diffraction measurements that fluorescence suppression strongly improves the signal over background ratio and that low intensity data is not affected by a neighbouring saturated signal. Therefore, this detector is the best available instrument for modern diffraction and diffuse scattering measurements (Weber *et al.*, 2008 ▶).

Developments in the near future are expected to include faster framing, smaller pixels and thicker and high-*Z* sensors for improved detection efficiency at higher energies.

## Figures and Tables

**Table 1:** Principal properties of the PILATUS detector system

ROC design	0.25 µm CMOS (radiation tolerant layout)
Pixel size	172 µm × 172 µm
Counter depth	1048574 (20 bit counter pixel^−1^)
Threshold fine adjustment	6-bit DAC pixel^−1^
Readout clock frequency	66 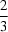 MHz
ROC array size	60 × 97 = 5820 pixel
ROC size	17.54 mm × 10.45 mm
Module array size	487 × 195 = 94965 pixel
Module active area	83.78 mm × 33.56 mm (continuous)
Module readout time	2.85 ms

**Table 2:** Overall energy resolution of a trimmed PILATUS module for 8 keV X-rays and three different CSA gain settings

	Low gain	Medium gain	High gain
FWHM (eV)	1082 ± 3	1038 ± 9	863 ± 4

## Footnotes

1 Each readout channel is electrically connected to its corresponding detecting element through a microscopic indium ball.

2 Sharing of signal charge between neighbouring pixels (*cf.* §3.2).

3 Δ*E*/*E* of the incident beam is < 2 × 10^−4^ [Si(111)] (Patterson *et al.*, 2005 ▶).

4 Two rows or columns of larger sensor pixels span the gaps between ROCs, thus there is no dead area between chips. The registered counts of two of these larger pixels are redistributed by software into three normal-sized pixels by introducing a virtual pixel in between (Kraft *et al.*, 2009 ▶).

5 The intrinsic threshold dispersion of a module is significantly reduced by pixel-to-pixel threshold trimming (Eikenberry *et al.*, 2003 ▶; Kraft *et al.*, 2009 ▶).

6 Note that these are distorted with respect to the reciprocal-space coordinates, unless a proper coordinate transformation is carried out, although this is unnecessary for the present purpose.
